# Impact of cannabis consumption on the course of bipolar disorder

**DOI:** 10.1192/j.eurpsy.2021.1507

**Published:** 2021-08-13

**Authors:** S. Brahim, M.H. Aoun, R. Boukhchina, S. Ben Frej, M. Henia, L. Zarrouk

**Affiliations:** 1 Psychiatry, University Hospital of Mahdia, Tunisia., chebba, Tunisia; 2 Department Of Psychiatry, University Hospital Of Mahdia, Tunisia., Psychiatry, Mahdia, Tunisia

**Keywords:** Cannabis, Addiction, bipolar disorder, abuse

## Abstract

**Introduction:**

Although one third of patients with Bipolar Disorder have an addiction to Cannabis or an abused consumption, the interaction between cannabis use and bipolar disorder remains controversial.

**Objectives:**

To evaluate the use of cannabis among patients with Bipolar Disorder and to compare the socio-demographic and clinical characteristics between patients who are consumers and non-consumers.

**Methods:**

This is a retrospective, descriptive study including all patients treated for type I bipolar disorder in the psychiatric department of Tahar Sfar Hospital of Mahdia (Tunisia). In addition to socio-demographic and clinical characteristics, we collected data on cannabis use (age at first consumption and frequency of consumption).

**Results:**

Our study population consisted of 84 male patients followed for bipolar I disorder. The mean age was 36.8 ± 11.3 years. Among these patients, 23 (27.8%) had regular cannabis use. The average age at first consumption was 21.6 ± 7.2 years. Bipolar patients with regular cannabis consumption had an earlier age of onset of the disorder (p = 0.02). They had higher numbers of manic episodes (p = 0.05), higher number of manic episodes with severe intensity (p = 0.04), higher number of manic episodes with mixed characteristics(p = 0.04), a higher number of hospitalizations (p = 0.01) with longer hospital stays (p = 0.02).

**Conclusions:**

Cannabis use among patients with type 1 bipolar disorder is associated with an unfavorable course of the disorder. Early diagnosis and appropriate management of this comorbidity seem to be essential for improving the prognosis of bipolar disorder.

